# Right versus left atrial pacing in patients with sick sinus syndrome and paroxysmal atrial fibrillation (Riverleft study): study protocol for randomized controlled trial

**DOI:** 10.1186/1745-6215-15-445

**Published:** 2014-11-17

**Authors:** Tanwier TTK Ramdjan, Lisette JME van der Does, Paul Knops, Jan CJ Res, Natasja MS de Groot

**Affiliations:** Unit translational electrophysiology, Department of Cardiology, Erasmus Medical Center, ‘s-Gravendijkwal 230, 3015 CE Rotterdam, Netherlands

**Keywords:** Sick sinus syndrome, Drug-induced bradycardia, Paroxysmal atrial fibrillation, Brady-tachy syndrome, Preventive pacing, Coronary sinus pacing, Right atrial appendage pacing, Remote monitoring, Atrial burden

## Abstract

**Background:**

The incidence of sick sinus syndrome will increase due to population ageing. Consequently, this will result in an increase in the number of pacemaker implantations. The atrial lead is usually implanted in the right atrial appendage, but this position may be ineffective for prevention of atrial fibrillation. It has been suggested that pacing distally in the coronary sinus might be more successful in preventing atrial fibrillation episodes. The aim of this trial is to study the efficacy of distal coronary sinus versus right atrial appendage pacing in preventing atrial fibrillation episodes in patients with sick sinus syndrome.

**Methods/Design:**

This study is designed as a multicenter, randomized controlled trial. Patients with sick sinus syndrome and at least one atrial fibrillation episode of 30 seconds or more in the six months before recruitment will be eligible for participation in this study.

All participants will be randomized between pacing distally in the coronary sinus and right atrial appendage. Randomization is stratified for all participating centers. Conventional dual-chamber pacemakers with advanced home monitoring functionality will be implanted. The ventricular lead will be implanted in the right ventricular apex. The first three months of the 36-month follow-up period are considered as run-in time. During the pre-randomization visit and follow-up, an interview, electrocardiogram and pacemaker assessment will be performed, prescribed antiarrhythmic medication will be reviewed and patients will be asked to complete an SF-36 questionnaire. An echocardiographic examination will be conducted in the pre-randomization phase and at the end of each follow-up year. Home monitoring will be used to send daily reports in case of atrial fibrillation episodes.

**Discussion:**

This randomized controlled trial is the first in which home monitoring will be used to compare atrial fibrillation recurrences between pacing in the distal coronary sinus or right atrial appendage. Home monitoring gives the opportunity to accurately detect atrial fibrillation episodes and to study characteristics of atrial fibrillation episodes. Should distal coronary sinus pacing significantly diminish atrial fibrillation recurrences, this study will redefine the preferential location of an atrial lead for preventive pacing.

**Trial registration:**

Current Controlled Trials ISRCTN65911661, registered on 8 July 2013.

## Background

Sinus node disease is defined as an intrinsic, symptomatic form of sinus node dysfunction and was first described by Wenckebach in 1923. It is also known as the sick sinus syndrome (SSS) and develops at a mean age of 68 years [1–5].

It is expected that the incidence of SSS will continue to increase due to ageing of the population, as impulse formation within the sinus node declines over time [6]. This may be caused by destruction of the sinus node itself or ischemia, inflammation or degeneration of the surrounding nerves or ganglia. If more than 90% of the sinus node tissue is damaged, SSS develops. However, sinus node dysfunction may also have extrinsic causes including antiarrhythmic drug usage, electrolyte disturbances, hypothermia, hypothyroidism, increased intracranial pressure and excessive vagal tone [6]. Sinus node disease accounts at present for approximately 35 to 45% of the pacemaker implantations in Europe and the United States and it is expected that the number of subjects requiring cardiac pacing will continue to rise in the coming decades [1, 6–9]. Within this group, 30% have symptomatic paroxysmal atrial fibrillation (PAF) [10]. The combination of sinus bradycardia or sinoatrial block and paroxysms of atrial fibrillation (AF) is also known as the brady-tachy syndrome [1, 4]. AF is associated with severe implications including heart failure and stroke; 20% of all strokes can be attributed to AF and patients with AF have a five-fold higher risk of stroke [11].

A meta-analysis by Coplen *et al*. revealed that only 50% of the PAF patients who were treated with anti-arrhythmic drugs remained in sinus rhythm (SR) during a one-year follow-up period [12]. In addition, the heart rate of SSS patients, who already have a sinus bradycardia, will become even lower when using antiarrhythmic drugs for symptomatic episodes of AF. In the PAF study, atrioventricular junctional (AVJ) ablation with DDD pacemaker implantation was compared with pharmacological treatment [13]. Quality of life scores were higher in the non-pharmacological group and these patients were also less symptomatic.

However, AVJ junctional ablation is often considered the final step of AF therapy due to its irreversible nature. Chen *et al*. proposed that catheter ablation of AF can improve sinus node dysfunction, since 95% of the patients did not have a pacemaker indication after the procedure [14]. However, these patients were relatively young and not all patients with severe symptomatic SSS may benefit from this approach. Therefore, preventive pacing could be an appropriate treatment modality for this group of patients.

The aim of the ‘Right versus Left Atrial Pacing in Patients with Sick Sinus Syndrome and Paroxysmal Atrial Fibrillation’ (Riverleft) trial is to study the efficacy of distal coronary sinus (dCS) versus right atrial appendage (RAA) pacing in preventing AF recurrences in patients with SSS and PAF. Characteristics of AF episodes, such as the number and duration of recurrent AF episodes, will be examined and compared between both study arms. The selected pacemaker (Evia DR-T or Eluna DR-T, Biotronik, SE & Co. KG) for the Riverleft study is equipped with home monitoring (HM) technology, which makes continuous assessment of cardiac rhythm feasible [15, 16].

## Methods/Design

### Study design

**Figure 1:**
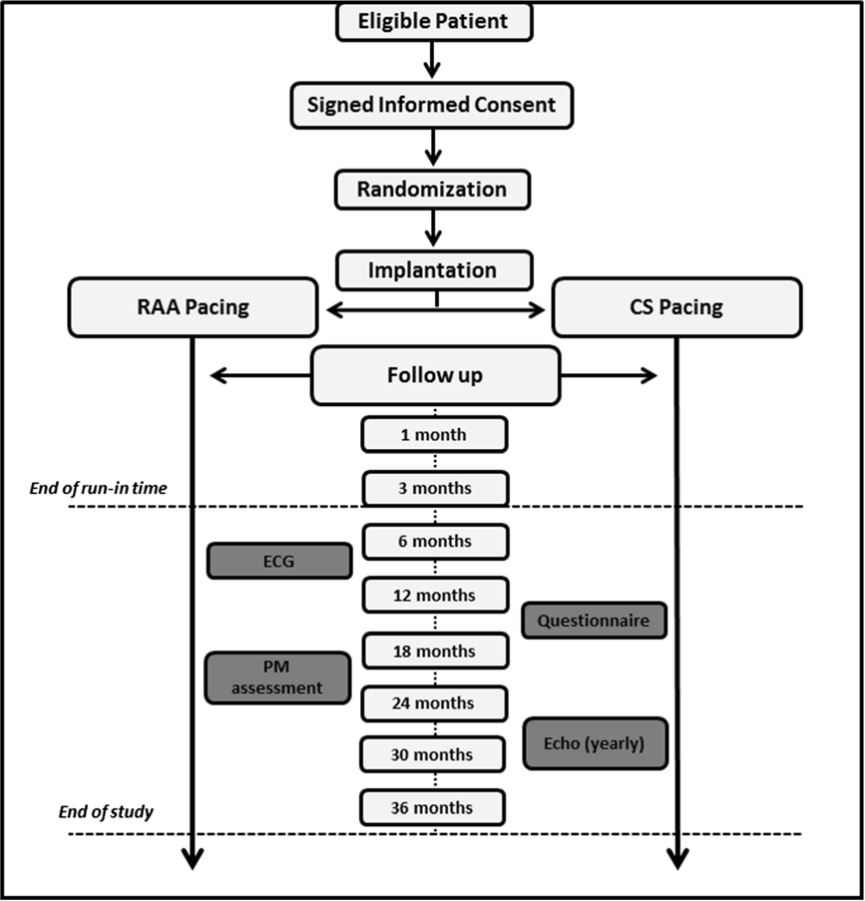
**Overview of the Riverleft study design.** Eligible patients will be informed about the study and asked to participate. Signed informed consent will be obtained prior to enrollment. In the next phase, the participant will be randomized between right atrial appendage (RAA) pacing and distal coronary sinus (dCS) pacing. A three-month run-in time will start after implantation. Adjustments to pacemaker settings and prescribed medication can be made without any consequences for the study. After finishing the run-in period, adjustments to pacemaker (PM) settings or prescribed drugs are only allowed if necessary. Electrocardiogram (ECG) will be made at each visit, an echocardiogram et the end of each follow-up year. The study will end for each participant after a 36-month follow-up period.

Riverleft is a multicenter, randomized controlled trial with an inclusion period of approximately two years and a follow-up period of three years for each enrolled patient. An overview of the Riverleft study design is given in Figure 1.

All participating centers will have an appointed local investigator who will be responsible for appropriate inclusion, implantation and follow-up. Furthermore, all centers will have an independent cardiologist, who patients with questions regarding the Riverleft trial can contact.

### Study population

Patients of 18 years or older, with either intrinsic or extrinsic (antiarrhythmic drug-induced) SSS and at least one PAF episode of 30 seconds or more within six months before enrollment, will be eligible to participate in the study. PAF episodes will be assessed by 24-hour Holter recording, preceding enrollment.

All patients with a clinically estimated life expectancy of five years or less or mental and/or physical inability to accomplish the follow-up period, will be excluded from participation in the Riverleft study. Left ventricular ejection fraction of 40% or less, malignancies and congenital heart defects are also exclusion criteria. Furthermore, chronic obstructive pulmonary disease classified as GOLD 4 and renal disease resulting in a glomerular filtration rate of ≤30 mL/min or creatinine value of ≥250 umol/L will prevent candidates from participation. The local investigator will only enroll eligible subjects after a signed informed consent form (ICF) is obtained.

### Sample size

A total number of 300 patients is required to detect a difference with a power of 0.8 between both study arms in recurrences of PAF. To maintain statistical power in case of dropout rates as high as 10%, 330 participants will be enrolled.

### Primary outcomes

The main objective of this study is to determine the effect of dCS pacing compared to RAA pacing for the prevention of recurrent AF episodes. Total number of AF recurrences and time between recurrences will be assessed and compared between both groups.

### Secondary outcomes

The relationship between pacing site, reduction of AF episodes, quality of life, heart failure, cerebrovascular accident (CVA), number of cardioversions, frequency and duration of hospital admission and AF progression to persistent AF will be examined.

### Randomization

Enrolled patients will be added to a tailor-made website, which will automatically randomize participants to either the control group (RAA pacing) or experimental group (dCS pacing). Randomization is stratified for all participating medical centers.

### Study groups

The study consists of two study arms; an atrial lead will be positioned in RAA position (group I) or in the dCS (group 2). Crossover will not be feasible due to the design of the study. All participants in both groups will have a ventricular lead implanted in the right ventricular apex (RVA). A conventional dual chamber with HM functionalities will be implanted in all patients.

### Study phases

#### Pre-randomization

Patients who meet the inclusion criteria will be approached by the local investigator or nurse practitioner to explain the Riverleft study and to hand over a patient information form. Candidates will be asked to participate in this study after a one-week reflection period. The nurse practitioner will call back or visit the candidate if the patient is hospitalized, and will obtain a signed ICF. Due to the obligation to give the patient a reflection period of seven days, pacemaker implantations in an acute setting cannot be enrolled in this study.

Eligible candidates, who agreed to participate in the study, will be scheduled for a visit to a cardiologist. During the intake visit, the patient will be asked to complete a health survey questionnaire (SF-36). Further examination includes an electrocardiogram (ECG) and cardiac echocardiography. Left atrial size will be determined in long-axis parasternal view and apical two-chamber view. In addition, left ventricular end systolic and left ventricular end diastolic diameter will be measured and diastolic function will be assessed by computing the ratio of early to late diastolic filling, the E/A ratio.

Medical records will be reviewed to determine the moment of SSS and PAF onset, to document accompanying symptoms during an AF episode and to assess risk factors, prescribed antiarrhythmic drugs and major events which are defined as CVA’s, transient ischemic attacks (TIA) or myocardial infarction. Practical and technological aspects of the selected pacemaker and HM will be explained by the pacemaker technician.

#### Implantation

The atrial lead will be placed in either the RAA (Solia, Biotronik, SE & Co. KG, Berlin, Germany) or dCS (SelectSecure 3830, Medtronic, Inc or Optisense Optim, St. Jude Medical, Inc or Solia, Biotronik, SE & Co. KG, Berlin, Germany). A SelectSecure 3830 will be positioned in the CS by using a steerable sheath (Steerable attain, C304-L6905, Medtronic Inc., Minneapolis, Minnesota, USA). Optimal lead position is determined by continuous monitoring of sensing and threshold and far field R wave (FFRW) values during slow retraction of the sheath. The tip of the steerable sheet will be bent and the lead will be screwed into the wall of the CS. Next, the steerable sheath will be removed completely. Implantation of an Optisense Optim lead (St. Jude Medical, St, Paul, Minnesota, USA), with a short tip-to-ring distance, will be considered in the event of unsatisfactory high levels of FFRW sensing. The investigators haven chosen screw-in leads in order to prevent CS lead displacements and to prevent high levels of atrial thresholds in the CS position as much as possible. A Solia screw-in lead in the CS position will be considered in the event of limited availability of a SelectSecure 3830 or Optisense Optim in the participating centre. A ventricular lead (Solia, Biotronik, SE & Co. KG, Berlin, Germany) is implanted in the RVA position. If this is not possible, the right ventricular outflow tract (RVOT) will be chosen as the alternative pacing site. Lead positioning is confirmed by using fluoroscopy. The implanting specialist will be advised to implant in the RVA or RVOT, in case of possible disparity in implanted numbers of RVA or RVOT between both study arms.

Conventional dual chamber pacemakers (Evia DR-T or Eluna DR-T pacemaker, Biotronik, SE & Co. KG, Berlin, Germany) with HM functionalities will be implanted. The basic rate will be adjusted to 70 beats per minute. Sensor rate and atrioventricular settings will be adjusted according to optimal levels for each individual patient. The closed loop stimulation (CLS) algorithm will be switched on, unless indicated otherwise. Pacemaker settings are encrypted and stored on a USB flash drive (16 gigabyte SanDisk Cruzer Glide USB Flash Drive, SanDisk corporation, Milpitas, California, USA) and imported to a dedicated website.

#### Hospital discharge

The pacemaker technician will give instructions regarding HM and will hand over a CardioMessenger II-S (Biotronik, SE & Co. KG, Berlin, Germany). The CardioMessenger requires only one wall socket and presence of a global system for mobile communications network for the first line of communication. This device will be installed in the bedroom of the participant.

#### Wound inspection

Patients will visit the outpatient clinic one week after pacemaker implantation. The pacemaker pocket will be inspected and in addition, correct functioning of the pacemaker and HM will be evaluated.

#### Run-in time

A three-month run-in time is reserved to allow lead stabilization, optimize pacemaker settings and establish an effective drug regimen. AF episodes registered in the three-month run-in time will not be added to the total number of counted AF episodes. Local investigators are requested to keep pacemaker settings and drug regimen unchanged during the rest of the follow-up period. Unavoidable modifications may be performed but have to be reported.

#### Follow-up

The three year follow-up period will consist of eight outpatient clinical visits. During an outpatient clinical visit, the cardiologist or nurse practitioner will interview the patient and a SF-36 questionnaire will be completed. Drug regimen will be reviewed for changes outside the run-in time. Comparable to the pre-randomization phase, an ECG will be made. Echocardiographic examination will be conducted every year in order to perform the same measurements as described in the pre-randomization phase. The pacemaker technician will assess pacemaker performance, store reports in the pacemaker memory and, if necessary, modifications in the pacemaker settings on encrypted USB flash drive.

Alongside clinical visits, HM will give the investigators the opportunity to receive a report from the implanted pacemaker on a daily basis. The CardioMessenger will automatically receive data from the implanted pacemaker once a day and will send this dataset to the HM website (Biotronik, SE& Co. KG). This report will contain measured values of sensing, threshold, impedance and program settings including CLS.

**Figure 2:**
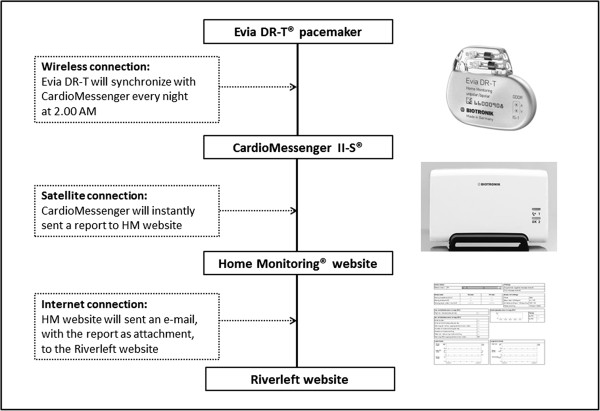
**The three modules of home monitoring and the connection with the Riverleft website.** An Evia DR-T pacemaker (Biotronik, SE & Co. KG) synchronizes with a CardioMessenger, preferably placed on the night table. The minimal distance of 20 cm and the maximum distance of 2 meters are mandatory for correct synchronizing between pacemaker and CardioMessenger. The CardioMessenger will send a report to the home monitoring (HM) center. Finally, an email, with the report attached, will be sent from the HM website to the Riverleft website.

In addition, HM will report on registered episodes of high atrial rate, number of mode switches and ongoing atrial episodes. The three modules of HM and the connection with the Riverleft website are explained in Figure 2. An Evia DR-T or Eluna DR-T pacemaker (Biotronik, SE & Co. KG) synchronizes with a CardioMessenger, preferably placed on the night table. The CardioMessenger will transmit the acquired data to the HM datacenter. Next, the tailor-made website will receive an email with the acquired data.

### Adverse events

According to the regulations of the medical ethics committee (MEC), it is compulsory to register all (serious) adverse events. Lead dislocation, pacemaker pocket infection, CVA, TIA and death are considered as (serious) adverse events.

### Dedicated website

We intended a nearly paperless study in order to make the study less vulnerable to errors. For this purpose, a tailor-made website was developed for the Riverleft study. Each participating medical center will have access to a dedicated part of the website. After enrollment and randomization of the participant on the website, obtained data from reviewed medical records and drug regimen, performed interview and ECG, conducted echocardiogram and SF-36 questionnaire will be completed on the website. The website will be completed after each outpatient clinical visit during the follow-up period. The follow-up status of all patients can be viewed instantly.

This website will also receive emails from the HM monitoring website in case of recurrences of AF. Reports on episodes of high atrial rate and number of mode switches will be imported and stored on the website. If necessary, pharmacological therapy will be modified or cardioversions will be scheduled. All adverse events will also be documented on the tailor-made website.

### Safety monitoring

Two committees and one monitor were appointed for patient safety purposes. A data safety monitoring board will review the study progress, make recommendations regarding established protocol and study progress and has the ability to stop the study due to significant benefits or adverse effects in one of the two study arms. This board has three members: an experienced cardiologist, statistician and a researcher. The clinical events committee will study all (serious) adverse events for a causal relationship between event and the Riverleft study. An independent cardiologist will evaluate protocol compliance and study progress by taking random samples in all participating centers.

### Ethics

The study protocol was approved in October 2012 by the MEC (2012-087) in the Erasmus Medical Center, Rotterdam, The Netherlands.

### Statistical analysis

This study is designed to compare recurrences of AF episodes between two treatment groups. Therefore the Fisher’s exact test will be used for this categorical data to assess the difference in AF recurrence. Continuous data regarding duration and total number of AF recurrences will be analyzed by the Student’s t-test. Kaplan-Meier curves will be constructed to compare event-free survival between both groups. Secondary outcomes will be analyzed by a multivariate analysis. A *P* value of 0.05 is considered to be statistically significant.

## Discussion

This randomized controlled trial is the first of its kind in which HM will be used to compare AF episodes, total number and duration of AF recurrences between RAA pacing and dCS pacing.

### Mechanism of atrial fibrillation in patients with sick sinus syndrome

Local conduction abnormalities may be involved in the pathogenesis of AF. Atrial conduction delay was found in 68% of patients undergoing pacemaker implantation for sinus node disease [17]. In order to study heterogeneity in conduction in SSS patients (with or without AF) electrophysiological studies were conducted during SR to determine the degree of fractionation in the right atrium (RA) [18–23]. Fractionation indicates asynchronous activation of atrial tissue and extensive fractionation may predispose to the development of PAF. Fractionated electrograms were recorded in more than 80% of SSS patients with PAF. Compared to SSS patients without AF, fractionation was more extensive in patients with AF [18, 19]. The spatial distribution of fractionated atrial electrograms in the RA was assessed in a subsequent study. In SSS patients with paroxysms of AF, fractionated electrograms were recorded diffusely from the higher, middle and lower part of the RA. In contrast, fractionated electrograms in SSS patients without PAF were predominantly recorded in the high RA. Hence, these findings suggest that episodes of AF in SSS patients are associated with widespread electrophysiological alterations in the RA [20]. Next, the relationship between fractionated electrograms and inducibility of AF was studied in SSS patients; inducibility of AF was related to the incidence and severity of fractionation [21, 22]. In addition, fractionated electrograms were more frequently recorded in older SSS patients with PAF. These findings may explain why older SSS patients are more prone to developing PAF [23].

### Mechanism of preventive pacing

The number of AF episodes in SSS patients may be reduced by pacing in the atria. Preventive pacing inhibits AF by averting sinus bradycardia or by suppressing premature beats. AF initiation by premature beats was studied by Duytschaever *et al*. [24]. In the goat model of AF, the investigators revealed that preventive pacing reduced the window of inducibility by preventing the occurrence of late premature beats. Preventive pacing was more effective when close to areas of conduction block and remote from the origin of premature beats. However, preventive pacing may be difficult as there may be multiple areas of conduction block in patients with diseased atria. In addition, single or multiple premature beats can originate from different sites within the right or left atrium [24].

Inhibition of AF induction by pacing from the high right atrium (HRA) and dCS was examined during electrophysiological studies in 13 patients by Papageorgiou *et al*. [25]. Atrial extra stimuli were applied from the HRA during pacing from both the HRA and dCS. Stimulation from the HRA was often unable to suppress AF induction, but dCS pacing suppressed AF induction at the same coupling intervals. dCS pacing significantly prolonged the coupling interval of atrial extrastimuli at the posterior triangle of Koch. The investigators proposed that dCS pacing limits the induction of AF by diminishing prematurity of atrial extrastimuli in the area of the posterior angle of Koch. This, in turn, might prevent local conduction delay and reentry [25]. In a study by Verlato *et al*. pacing from the interatrial septum (right above the CS ostium) only prevented development of persistent AF in patients with atrial conduction delay at the triangle of Koch [17]. Possibly, these patients are more susceptible to atrial ectopy originating in this area. However, pacing at the dCS may also prevent AF induction due to ectopic beats arising from other areas.

### Preferential pacing modalities and sites

The first results of pacing in the coronary sinus (CS), were published by Moss and Rivers in 1978 [26]. He reported on a 10-year experience of CS pacing in a total number of 50 patients. Five years after implantation, more than 75% of the patients had appropriate CS pacing. Recurrences of difficult-to-treat supraventricular and ventricular tachyarrhythmias were prevented by CS pacing in four patients. Despite the limited experience with cardiac pacing in the late sixties and early seventies, the investigators assumed that pacing would become a treatment modality for recurrent atrial tachyarrhythmias [26]. However, CS leads were difficult to implant and occasionally resulted in perforation of the CS. RAA pacing was therefore developed as an alternative site for CS pacing [27]. RAA pacing in SSS patients was associated with a high level of intra-atrial conduction disturbances as reflected by prolonged P-wave width and a high incidence of AF recurrences [28]. Saksena *et al*. combined RAA pacing with proximal CS pacing and named this dual-site (DS) pacing [29]. In this trial, 15 patients with drug-refractory AF and coexistence of bradyarrhythmia were enrolled. The DS configuration was used in the first three months after implantation. In the last three months, patients were switched to single-site RAA pacing. No AF recurrences were observed during DS pacing, compared to five recurrences in 12 patients with RAA stimulation (*P* =0.03). Hence, the results of DS pacing were promising and it appeared to be feasible, safe, effective and suitable for long-term application [29].

Next, the DAPPAF trial was designed to compare DS pacing with proximal coronary sinus (pCS) pacing. One recommendation made by these investigators in their published study protocol was to perform a future study in which pCS pacing would be compared with RAA pacing. The aim of future studies should be to determine if the expected positive effect is caused by DS pacing or proximal CS pacing alone. At that time, pCS pacing could not be compared with RAA pacing due to technical limitations. The RAA lead was connected to the negative connector of the bipolar adapter and the CS lead to the positive connector. A dual bipolar set-up could not be established with the use of the approved bipolar adapter. DS pacing could be switched to single-site RAA pacing by selecting unipolar pacing only. Therefore, the investigators were unable to program solely pCS pacing [30]. Enrolled patients in the Riverleft trial will receive a conventional dual-chamber pacemaker with one atrial and ventricular channel. No bipolar adapter is required to bifurcate the atrial stimulus. Therefore, technical requirements for the selected pacemaker and lead configuration are less extensive, compared to the studies conducted by Saksena *et al*. and Fitts *et al*. [29, 30].

Different pacing algorithms for prevention of atrial tachyarrhythmias were tested by Blanc *et al*. with little success; only a subgroup of patients with a low percentage of ventricular pacing benefitted from atrial pacing [31]. However, the atrial lead was implanted at the conventional site (RAA).

A substudy by Mirza *et al*. examined lead performance over time and safety in the pCS position [32]. No lead dislodgements or adverse events were reported and the group concluded that permanent CS pacing is safe and feasible, even though active lead fixation was not used in this study. It has been demonstrated that active fixation in the CS shows good stability and safety compared to passive fixation [33, 34]. Based on the assumption by Papageorgiou *et al*. [25] that dCS pacing diminishes prematurity of the atrial extra stimuli in the posterior triangle of Koch, Breuls *et al*. initiated a pilot study to compare dCS pacing with RAA pacing [35]. They demonstrated that left atrial stimulation tends to be more effective than right atrial stimulation in terms of reduction of AF episodes and improvement in quality of life in SSS patients with paroxysmal AF.

### Evaluation of atrial fibrillation episodes

Ricci *et al*. evaluated the capability of HM to detect AF episodes in 166 patients [15]. The investigators concluded that HM allows early AF detection which, in turn, can result in an earlier intervention by a clinician [15]. Feasibility and safety of HM was already acknowledged by ‘The Lumos-T Safely RedUceS RouTine Office Device Follow-up’ (TRUST) trial which included 1339 patients with an implantable cardioverter defibrillator. Participants were randomized in a 2:1 ratio to HM only or conventional outpatient clinic visits after 3, 6, 9, 12 and 15 months follow-up. Three and 15-month HM evaluations were performed in conjunction with a clinical visit of the patient. Scheduled and unscheduled clinic visits, morbidity and elapsed time between recorded event and clinical evaluation were assessed. Up to a 45% decrease of clinical pacemaker assessments was achieved with HM only evaluations, without affecting morbidity. Less than 15% of all HM only evaluations required unscheduled clinic visits. The TRUST investigators concluded that remote monitoring can be regarded as a safe alternative to conventional follow-up and allows a more rapid response after a recorded event [16]. However, the Heart Rhythm Society/ European Heart Rhythm Association expert consensus recommended that monitoring of cardiovascular implantable electronic devices should be accompanied by an outpatient clinic visit at least once a year [36]. No interval longer than six months is scheduled in the three-year follow-up period of the Riverleft trial. Use of remote monitoring was endorsed in the most recent guidelines on cardiac pacing and resynchronization therapy of the European Society of Cardiology [37].

### Study design

The Dutch Dual-site Right Atrial Pacing for Prevention of Atrial Fibrillation (DRAPPAF) trial randomized 26 patients with symptomatic drug-refractory AF between DS pacing and RAA pacing, but patients with underlying sinus node disease were not included [38]. At hospital discharge, patients were randomized to DS pacing or RAA pacing for a duration of six months. After this period, participants were crossed over to the other group. No patients in the DS pacing group required cardioversions or deteriorated to permanent AF, compared to four and one participants, respectively in the RAA pacing group [38]. In the earlier mentioned trial of Saksena *et al*., 15 patients with SSS and episodes of AF were crossed-over from DS pacing after 90 days to RAA pacing for a period of three months [29]. Consequently no run-in time was acknowledged and the efficacy of a pacing modality could have been over- or underestimated due to a carry-over effect. The Riverleft study differs from the DRAPPAF study and the study by Saksena *et al*. in the absence of a crossover design. Follow-up periods of six (DRAPPAF study) and 12 (Saksena *et al*.) months are, from our point of view, too short to assess the preventive effect of CS pacing and to compare this with conventional RAA pacing.

Registration of AF episodes in both studies could only be established by complaints of the participant or by patient-activated ECG recording. Due to integration of HM in the Riverleft study, symptomatic and asymptomatic AF episodes will be registered automatically. Therefore, the Riverleft investigators will be able to reveal a more reliable quantification of the AF burden in the two study groups.

### Limitations

Accurate AF detection depends on proper sensing and pacemaker programming. FFRW sensing could be a considerable problem if CS pacing is intended. Therefore, careful inspection of adequate atrial sensing (levels) and FFRW signals should be carried out during the implantation procedure and be confirmed at each follow-up visit. The patient will be excluded from further participation if there is a marginal low atrial sensing or persistent high amplitudes of FFRW signals.

HM will only send a triggered message after at least 20 mode switches a day. This restriction will limit the ability to detect the first onset of AF recurrence after pacemaker implantation. Besides mode switch registration, high atrial rate will be used to register AF episodes. In contrast to mode switches, one episode of high atrial rate will trigger a HM message. The disparity between both study arms for location of implanted ventricular lead, should be monitored carefully. A high number of implanted leads in the RVOT, could influence the outcome for the respective study arm.

## Trial status

Inclusion of eligible candidates in the Riverleft study has not yet started and is expected to start in November 2014.

## Abbreviations

AF: Atrial fibrillation
AVJ: Atrioventricular junction
CS: Coronary sinus
CLS: closed loop stimulation
CVA: Cerebrovascular accident
dCS: Distal coronary sinus
DS: Dual-site
DRAPPAF: Dual-site Right Atrial Pacing for Prevention of Atrial Fibrillation
ECG: Electrocardiogram
FFRW: Far field R wave
HM: Home monitoring
HRA: High right atrium
ICF: Informed consent form
MEC: Medical ethics committee
PAF: Paroxysmal atrial fibrillation
pCS: Proximal coronary sinus
PM: Pacemaker
RA: Right atrium
RAA: Right atrial appendage
RVA: Right ventricular apex
RVOT: Right ventricular outflow tract
SR: Sinus rhythm
SSS: Sick sinus syndrome
TIA: Transient ischemic attack
TRUST: The Lumos-T Safely RedUceS RouTine Office Device Follow-up trial.
